# Helicopter Inside Cabin Acoustic Evaluation: A Case Study—IAR PUMA 330

**DOI:** 10.3390/ijerph18189716

**Published:** 2021-09-15

**Authors:** Marius Deaconu, Grigore Cican, Adina-Cristina Toma, Luminița Ioana Drăgășanu

**Affiliations:** 1 National Research and Development Institute for Gas Turbines COMOTI, 220D Iuliu Maniu, 061126 Bucharest, Romania; marius.deaconu@comoti.ro (M.D.); adina.toma@comoti.ro (A.-C.T.); luminita.dragasanu@comoti.ro (L.I.D.); 2 Department of Aerospace Sciences, Faculty of Aerospace Engineering, Polytechnic University of Bucharest, 1-7 Polizu Street, 1, 011061 Bucharest, Romania

**Keywords:** helicopter cabin, noise levels, noise reduction, acoustic evaluation, IAR Puma 330

## Abstract

This paper presents an inside-cabin acoustic evaluation of the IAR PUMA 330 helicopter, manufactured by IAR S.A. Brasov. In this study, based on the acoustic assessment inside the helicopter, areas with high noise levels are identified. In this regard, several tests were carried out in accordance with the ISO 5129 standard. In the first stage of the assessment, a measurement campaign was performed to identify the acoustic leaks from the outside noise sources propagating inside the cabin (in the door area) and the acoustic attenuation of the helicopter structure. These tests were performed on the factory runway, with the helicopter in parked position (ground tests). During the ground tests, the helicopter engines were turned off. The tests consisted of placing two loudspeakers directed towards the helicopter door and generating pink noise. Inside the helicopter, the entire door frame opening was scanned with an intensity probe to identify acoustic leaks areas. The second assessment stage was to determine the areas of the cabin with the highest levels of noise. Within the measurement campaign, 16 microphones were placed inside the cabin, at the level of the passengers’ heads, arranged in seven zones. The tests were carried out with the helicopter engines started, staying at fixed point above the ground (hovering), and then a flight test, in which all the maneuvers necessary for the use of the helicopter were performed (in-flight tests). Based on the measurement results, it was possible to highlight the noise spectral components in each of the seven areas. The noise assessment revealed high noise levels inside the cabin, having as main noise sources the transmission gear and the door area, leading to the need for reducing the noise exposure for passengers and crew, thus the need to reduce noise levels inside the helicopter.

## 1. Introduction

Nowadays, noise pollution represents one of the main problems for aviation development [1] due to the need to heavily reduce the noise exposure of the areas adjacent to airports or heliports. It is known that the noise exposure to the crew and especially the passengers and the people around landing and take-off areas represents a great issue [2]. 

**Figure 1:**
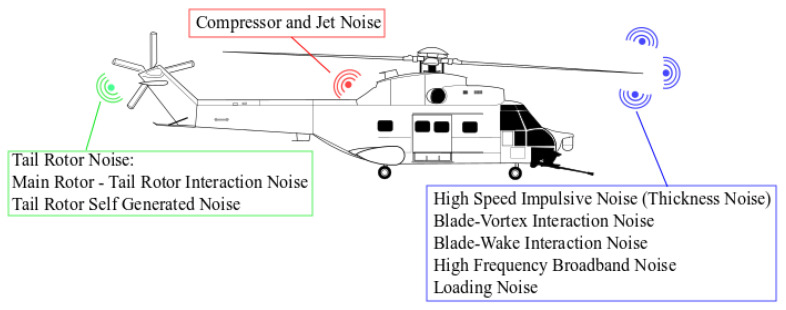
Helicopter noise sources, generation locations and their classification.

In terms of helicopter noise sources, the main sources are: rotor, anti-torque, engines, gear box, depending on flight condition, transmission gear, etc. [3] and are illustrated in Figure 1.

In a short review of the noise sources generated by the helicopter and connected to the present study, it is worth mentioning the thickness noise which is caused by the blade periodically displacing air during each revolution and is dependent only on the shape and motion of the blade. Generally, the thickness noise propagates in the plane of the rotor as well as the high-speed impulsive noise. In addition, the loading noise is another type of noise source which influences the inside-cabin noise. The loading noise is directed below the rotor and is caused by the acceleration of the force distribution on the air around the rotor blade passing through it. Another influence is the blade vortex interaction which is directed down and rearward and it occurs when a rotor blade passes within close proximity of the shed tip vortices from a previous blade [4,5].

From the frequency point of view, the most annoying noise for the human ear is the tail rotor noise due to its higher frequency which coincides with the band to which the human ear is most sensitive [6,7,8].

The inside-cabin noise levels depend on the flight conditions, maneuvers and the observer position inside the helicopter. All these combined influences are detailed by the joint work of Snecma, Airbus Helicopters, Sikorsky Aircraft, Bell Helicopter, Agusta Westland, Turbomeca, Marenco Swisshelicopter and the Research Centers: NASA, DLR, ONERA, JAXA in [9]. This comprehensive study is highlighting the fact that implementing a sophisticated noise reduction technology addressing one noise source may reduce the noise level in one flight condition; there may be, however, no change or in some cases increases in the noise levels in other flight conditions [9]. 

Helicopters with a good acoustic level are considered to be those which have 70 dB(A) in the interior, while those in which the noise exposure exceeds 85 dB(A) equivalent sound level are considered to be a potential risk [10].

The purpose of this paper is essentially to study the acoustic field inside the cabin, to determine the inside noise level, whether soundproofing structures are needed, and to determine to what frequency domain the structures must be designed.

It is very important to have the complete noise description inside the helicopter cabin, in order to know the exposure level and to find ways to improve the noise conditions.

A similar paper that approaches the acoustic evaluation of helicopters and particularly focuses on acoustical comfort improvement in helicopter cabins is presented in [11]. There are available, and already performed, different methods to characterize the inside-cabin noise, and also methods used to localize noise sources inside helicopter cabin from in-flight tests [12,13]. Several studies have been conducted regarding helicopter noise such as: acoustic performance measurements during flight for a Bell 206B helicopter [14], exterior noise level induced by two different rotor blades for two helicopters, R44 Clipper and Bell 206B [15]; and another study presents the results of measurements of noise generated by helicopter SA341H “Gazelle” at full throttle in the landing and take-off phase [16]. Here, the level of noise generated by helicopter type SA341H “Gazelle” was compared to the levels of noise generated by the Robinson R44 Clipper and Bell 206B helicopters. Nelson [17] presents acoustic measurements and data collected inside a U.S. Army Sikorsky UH-60 helicopter to analyze the inherent noise present during routine aeromedical transport.

Addressing a similar goal to our research study is the work performed by Eurocopter, the difference between that work and our research being the fact that they used a modelling methodology coupled with Nearfield Acoustical Holography measurements and geometrical acoustics [18].

The acoustic evaluation of the IAR PUMA 330 helicopter was realized following the ISO 5129 standard. For the acoustic measurement, 16 microphones were placed, according to standard, inside the cabin, at the passenger head level and the noise levels were measured during different maneuvers performed in flight. The noise level variations for each microphone were correlated with the maneuvers during the flight. Another stage consisted of performing tests with the helicopter on the ground with the engine turned off, the noise source being two loudspeakers placed near the helicopter with the purpose of identifying any acoustic leaks from door frames. Finally, conclusions are drawn regarding the level of noise and the inside characteristics of the cabin.

## 2. IAR PUMA 330 Helicopter Technical Description

**Figure 2:**
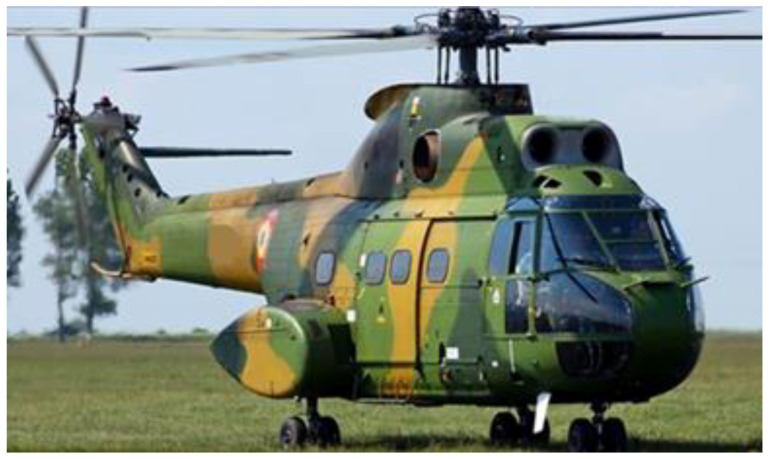
IAR PUMA 330 Helicopter.

IAR PUMA 330 helicopters, Figure 2, are built, maintained and upgraded by the IAR Company S.A. BRASOV [19]. According to the technical datasheet, the crew of this helicopter is composed of three members. The helicopter has a capacity of 16 passengers and a length of 18.22 m. The helicopter has a height of 5.14 m, having a rotor with a diameter of 15.08 m. The empty weight of such a helicopter is 3615 kg, with the take-off maximum of 7400 kg. The helicopter has two TURMO IV C turboshafts with a free turbine, 1,175,000 W each one. The helicopter reaches a maximum speed of 263 km/h and has a range of action of 550 kilometers without other supplementary tanks. The technical features include a 4800 m service cap and an ascending speed of 9.2 m/s [20]. IAR PUMA 330s are helicopters of the 1970s, built to the military specifications of the time when the rules for cab noise were more permissive.

## 3. Testing Procedures

The method used to determine the noise level inside the helicopter during the flight is given by the international ISO 5129 standard [21], which specifies that the measurements should determine the sound pressure level A-weighted and in 1/3 octave band. In addition, ISO 5129 specifies that the sound pressure levels must be measured at the head level of the passengers, but no passenger must be present during the tests. The measuring positions were chosen to determine the acoustic field in the passengers’ position and in the entire helicopter. The microphones were fixed with a metallic extension attached to the helicopter frames and a damping material was used as interface between microphone and metallic support to minimize the vibrations’ effects on the acoustic signals. The acoustic measurement was performed over the entire flight time. During the acoustic measurement, the helicopter was in a minimal configuration: without upholstery and chairs. During the flight test, only the crew and the acoustic team were present in the helicopter. The first step of the research was to identify the level of sound pressure inside the cabin of the helicopter using the actual soundproofing structures used by IAR Brasov and the acoustic leaks caused by the imperfections of door tightness. For the noise leak detection, the method specified in SR EN ISO 9614-2/2000 [22] was used; a method that is based on mapping sound intensity over the area of interest.

## 4. Measurement Campaign

The purpose of the inside acoustic evaluation of the IAR 330 cabin was to identify the locations with high noise levels and the cabin’s overall noise level. During the helicopter’s functioning, inside the cabin, the main source of noise is represented by the transmission box lid, situated close to the passengers’ location. Other sensitive locations that can produce a lot of noise are the door areas due to their sealing system. The noise emitted by the exhaust engines (situated above both doors), combined with the aeroacoustics noise, come through the doors’ weather-strips. It must be mentioned that the turboshaft engines are fixed on a rigid metal plate that has no physical contact with the cabin indoors.

For noise mapping and sound field characterization, the measurement campaign consists of two steps: ground tests (acoustic measurements with helicopter engines and auxiliary units off) and in-flight tests, one in hovering mode and one performing all the maneuvers necessary for the use of the helicopter.

### 4.1. Flight Tests

**Figure 3:**
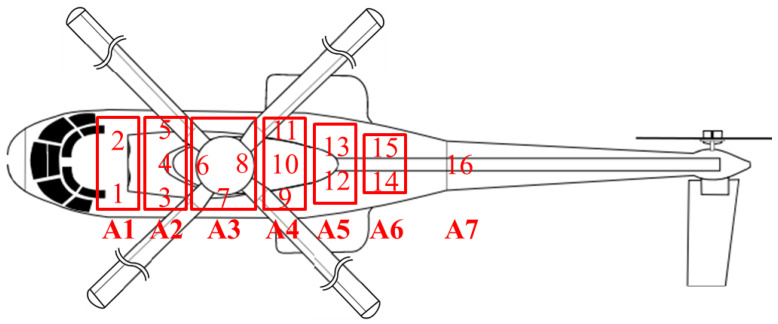
The helicopter cabin divided in 7 areas (A1 to A7) and microphone positions from 1 to 16.

**Figure 4:**
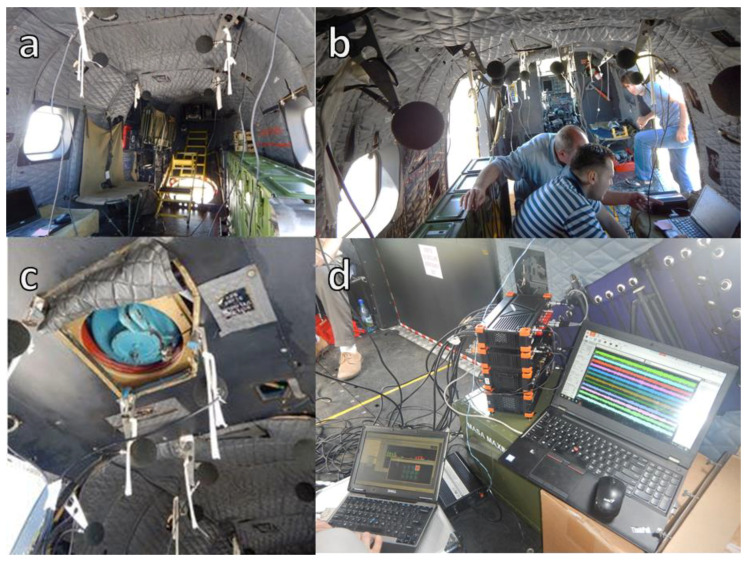
The locations of the microphones in the helicopter and the measurement equipment: (**a**) microphones from A5, A6, A7; (**b**) microphones from A1, A2, A3, A4; (**c**) microphones under transmission gear A3; (**d**) measurement equipment.

The inside cabin acoustic field was measured by mounting 16 microphones in different locations of the helicopter as is presented in Figure 3. The microphone grid was composed from 16 diffuse field microphones 40AQ type with preamplifier 26CA, which were mounted on special metallic supports, attached to the helicopter frames according to Figure 3. The acoustic signals were recorded with the multichannel acquisition system Sirius from DeweSoft using a sampling frequency of 50 ks/s (kilosamples per second). The calibration of the measurement channels was performed with the acoustic calibrator 42AB GRAS which generates at the frequency of 1 kHz an amplitude of 10.02 Pa (114 dB (ref. 2.0 × 10^−5^ Pa)). The helicopter cabin was divided in 7 regions corresponding to the structural frames on which were placed the microphone holders as can be seen in Figure 4. 

For the turbo engines influence, areas 1 and 2 were designated, area 3 for transmission gear, area 4 for the combined noise emitted by the transmission gear and exhaust noise, and areas 5, 6 and 7 to identify spectral components of other noise sources.

The flight test consisted in performing the following phases: engines start, hovering, hovering turn, hovering—forward, sidewards, rearward flight, turns, climb, descent.

### 4.2. Ground Tests

The purpose of the tests performed on the ground was to identify the acoustic leaks from the doors’ weather-strips and to determine the acoustic attenuation of the helicopter side structure (IL—insertion loss) with the current acoustic insulation solution of the IAR. The acoustic tests from the ground were conducted with the helicopter in flight configuration without the engines or other components working. The helicopter was placed on the IAR Brasov runway, with no other acoustic sources near to it during the measurements.

Considering the great distances from the closest nearby buildings, it was considered that during tests the free field condition was reached, so no influencing reflections were considered. At 1.5 m from the helicopter fuselage and 5 m from the loudspeakers, four 40AE microphones were mounted at 1.7 m height from the ground, oriented to loudspeakers. The outside microphones were used to check if the acoustic field generated by the two loudspeakers was diffuse.

Three 40AQ microphones were mounted inside the helicopter, one in the front area of the helicopter, the second one in the middle and the third one at the back. Based on the average sound pressure levels calculated based on the acoustic signals recorded with the outside microphones and the average sound level from the inside microphones, the insertion loss (IL) was calculated.

Before the field measurement campaign, an acoustic evaluation field was performed in the anechoic chamber [23] to determine the number of loudspeakers, their positions and at what distance should be used to obtain a diffuse field. The anechoic room volume was 1200 m^3^ with 15 × 10 × 8 m, wall absorption coefficient was 99% in frequency range of 50 Hz up to 20,000 Hz.

**Figure 5:**
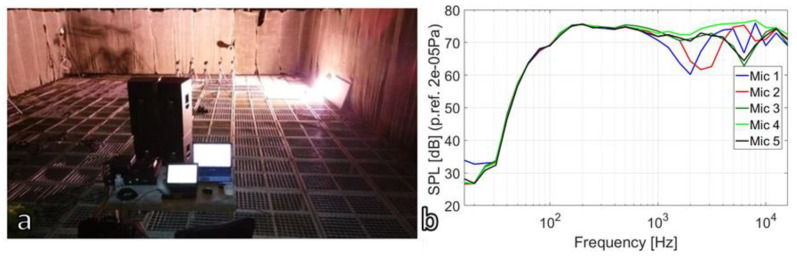
Diffuse field measurement set-up in anechoic chamber and obtained acoustic spectra: (**a**) microphones linear array and loudspeakers position in anechoic chamber; (**b**) averaged noise spectra in each microphone.

For these tests four HK Audio Linear L5 112 F loudspeakers and one power unit LD Systems DP2400X were used. Figure 5 presents one configuration of loudspeaker positioning during the anechoic measurements and the obtained noise spectra in each microphone.

Following the tests, it was found that the use of two loudspeakers rotated in different directions by about five degrees can generate a diffuse acoustic field at 5 m.

The spectral analysis presented in Figure 5 highlights that in the frequency domain of 50–1 kHz, the amplitude spectra of the five microphones have a variation of maximum 1.5 dB, while after 1 kHz the obtained differences between microphones reaches almost 10 dB, highlighting that a diffuse field in a specific frequency domain could be obtained using four loudspeakers.

**Figure 6:**
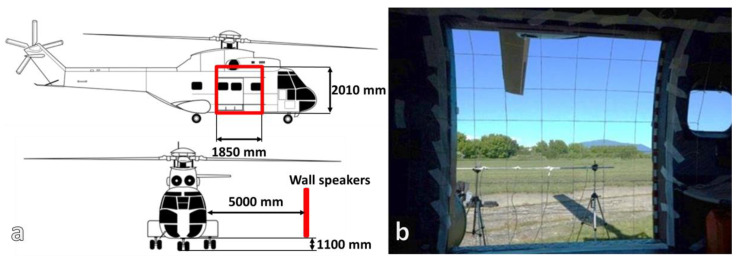
IAR Puma 330 acoustic intensity measurements set-up: (**a**) loudspeakers location, (**b**) outside microphones location and the grid for intensity scanning).

In parallel to these IL tests, acoustic intensity measurements were performed on the helicopter door area, Figure 6.

During flight, this area of the door is exposed from the outside of the helicopter to high levels of noise especially that caused by engine exhaust. The door area was divided into 81 surfaces and scanned with the 50AI GRAS intensity probe. The averaging time for each measurement point was 15 s. On the right side of Figure 6 the outside microphones can be observed with which the diffusion of the acoustic field was controlled and monitored.

## 5. Experimental Results

### 5.1. Flight Results

Flight results are presented both in the time and frequency domain for the entire duration of the flight. Variation of the overall noise level highlights the influences of the flight conditions. To have an overview of the noise inside the helicopter, an averaged acoustic pressure level for each researched area was calculated according to the standard ISO 3746 [24] using the following equation:

(1) $$\underline{L_{pA}^{\prime }}=10\;lg\;lg\;\left[\frac{1}{N}\overset{N}{\underset{i}{\sum }}10^{0.1L_{pAi}^{\prime }}\right]\;\;\left[dB\right]$$

where $\underline{L_{pA}^{\prime }}$ is the average A-weighted sound pressure level, in decibels, with the functioning helicopter; $L_{pAi}^{\prime }$ represents the A-weighted sound pressure level measured in *i* position of the microphone in decibels; *N* is the number of microphone positions. 

**Figure 7:**
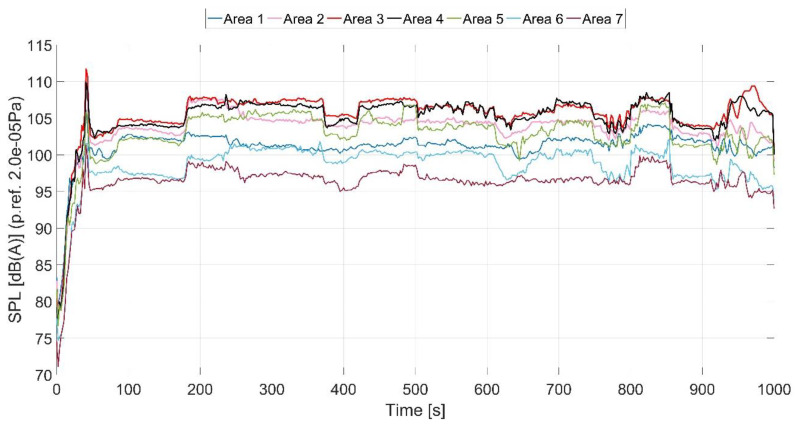
Noise variation in time in each measured area.

In Figure 7 the overall averaged noise levels’ variation in time is presented.

From Figure 7 which shows the variation of noise over time, it is observed that the areas with the highest noise level are 3 and 4; the noise level decreases in the helicopter’s other areas.

**Figure 8:**
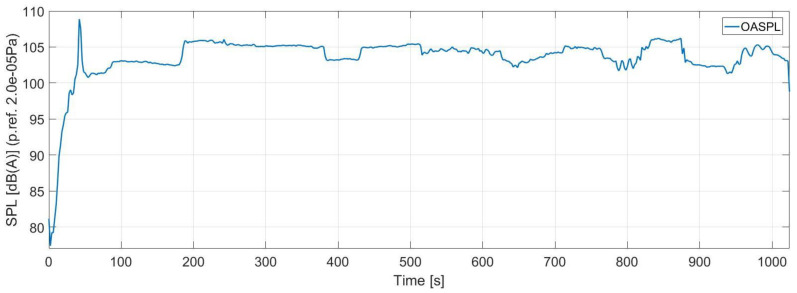
Noise variation for entire helicopter during the flight.

The overall noise level for the entire helicopter was computed based on the averaged noise curves from Figure 7, presented in Figure 8.

**Figure 9:**
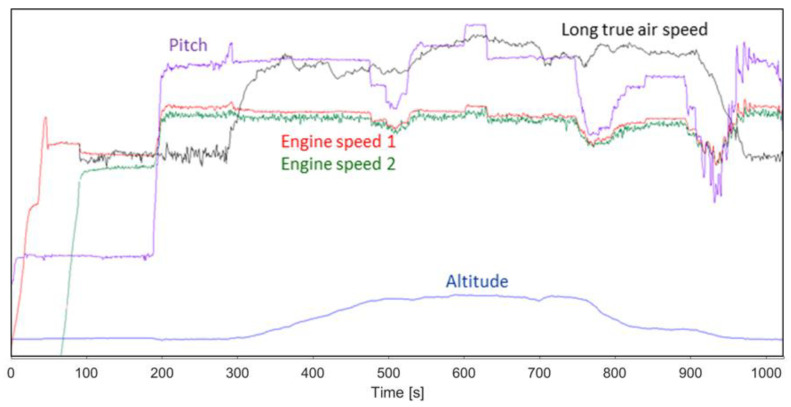
Helicopter flight data over the entire flight.

The overall noise level on the entire helicopter calculated according to the above relationship indicates a noise peak reaching 108 dB(A) during start-up, after which the noise level stabilizes to 103 dB(A) with variations depending on the flight conditions as presented in Figure 9. The range of the helicopter flight parameters are presented in Table 1.

During the flight the helicopter performed most of the maneuvers as can be seen from the flight data. By comparing the overall noise variation and the flight data, one can observe a correlation between the two sets of data. The starting of the first engine produces a noise peak with the biggest amplitude in time. The noise is related for the most part to the propeller pitch which influences the engines’ speed. So, a higher pitch leads to an increase of the engine speed, which has as effect an increase of the noise due to the bigger force in gear. Decreasing the pitch leads to a need of lower force from the free engine which leads to a lower engine speed and to a lower noise level.

**Figure 10:**
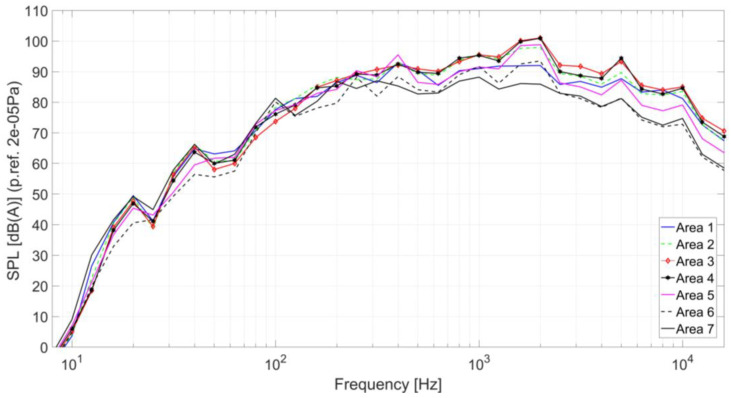
Averaged noise spectra in 1/3 octave bands for each measured area.

To observe tonal components and especially the values in the critical frequency domain for the human ear, a spectral analysis of the noise is required. Thus, the spectral analyses of the acoustic signals in each microphone are presented in the following figures. The acoustic signals during the flight were processed by using 1/3 octave band analysis resulting in an averaged spectrum for the entire flight period. Figure 10 presents the averaged noise spectra for each area. The averaged noise spectra for the areas 3 and 4 indicate that the amplitudes of all frequency bands are significantly higher than the other areas, especially in the frequency domain of 400–5000 Hz. Under 400 Hz the noise is generated mainly by the vibration of the helicopter structure and due to the big wavelengths, the noise differences between the areas are small. 

Over the frequency of 400 Hz differences between the areas start to be higher due to the fact that the acoustic sources are generated locally. 

Table 2 presents the overall noise level resulting from the acoustic spectra presented in Figure 10. The highest noise level is measured in Area 3, which is situated below the transmission gear, followed by the adjacent areas. It can be noticed that the noise level decreases by more than 9 dB to the back of the helicopter. The differences of the overall noise levels presented in Table 2 are highlighted using color code for each zone where the red one has high amplitude and the dark green is the lowest one.

**Figure 11:**
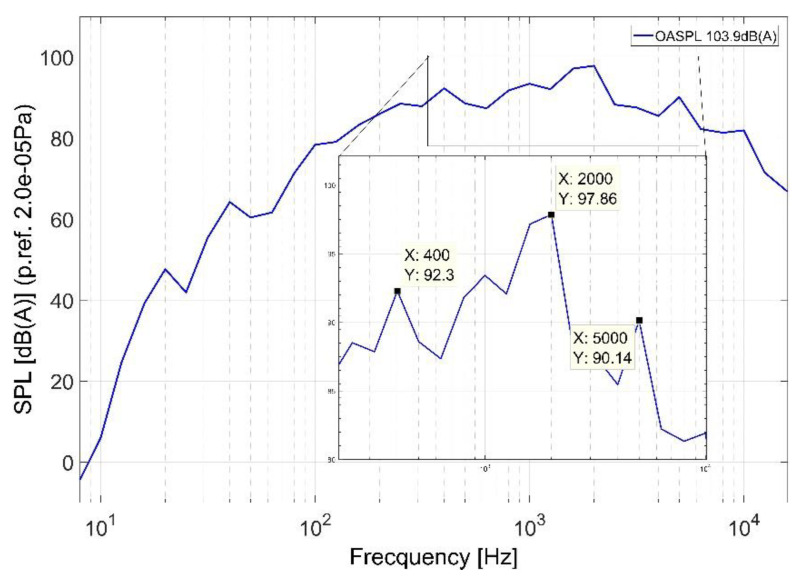
The average acoustic spectrum across the helicopter with maximum peak sounds at 400 Hz, 1 kHz, 2 kHz and 5 kHz.

Based on the spectral analysis presented in Figure 10, the average sound spectra were calculated for the entire helicopter, by using Equation (1). The average sound spectrum of the entire helicopter, presented in Figure 11, reveals several noise peaks at 400 Hz, 1 kHz, 2 kHz and 5 kHz. It must be mentioned that the peak from 2 kHz, a frequency where the human ear has an amplification of +1.2 dB (spectral weighting adjustment factor must be applied when converting between the weightings) [25], leads to an increase of the acoustic discomfort.

**Figure 12:**
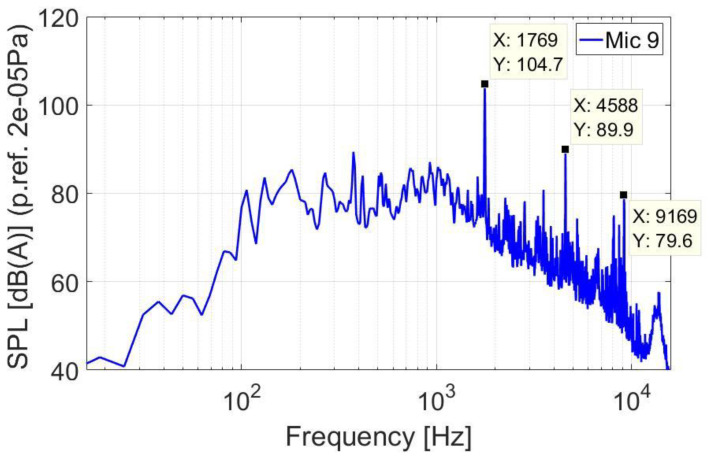
Instantaneous FFT spectrum of the highest noise level measured in microphone 9.

Figure 12 presents the FFT (Fast Fourier Transform) spectral analysis corresponding to the time step when the noise in the cabin was the highest. This level was recorded in area 3 by microphone 9 when an overall level of 112 dB(A) was measured, and the biggest spectral amplitude was at 1.7 kHz component with a value of 104.7 dB(A). 

**Figure 13:**
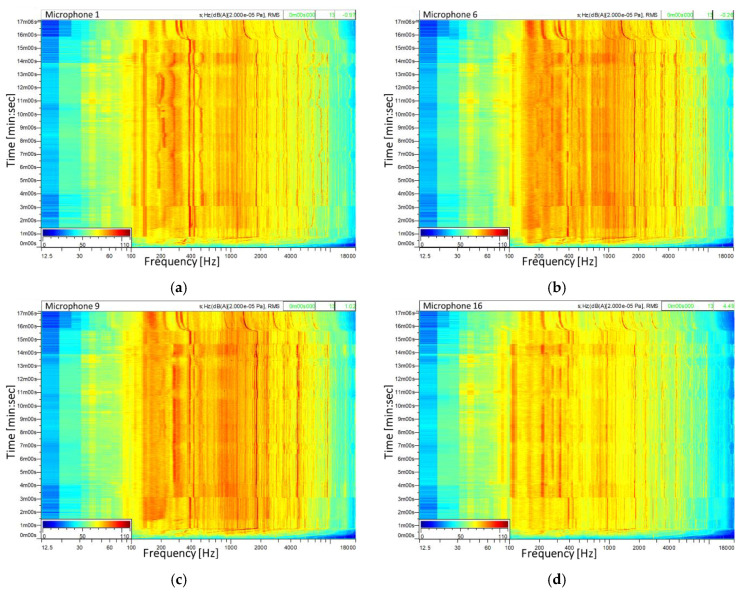
FFT analysis in time domain of the noise signals from: (**a**) microphone 1, (**b**) microphone 6, (**c**) microphone 9, (**d**) microphone 16.

For a better representation of the spectral components in time, Figure 13 presents a FFT analysis in the time domain for the entire flight time, for multiple noise signals. The time domain analysis has been performed with a spectral resolution Δ*f* = 25 Hz, time step *t_s_ =* 1 s, data blocks overlap of 70%. The results are presented as sonograms where the *X* axis represents the frequency, the *Y* axis represents the time and the color codes represent the sound pressure level amplitude. 

As can be observed from Figure 13a, at around 400 Hz two tonal components are identified which are generated by the speed of the two turbo shaft engines, the differences in frequency being produced by the different operating speeds of these two. The spectral components of the engines’ speed have the highest amplitudes in microphone 1 because the engines are located above it. 

The signals of microphones 6 and 9 highlight a broadband noise in the frequency range of 150–2000 Hz with strong tonal components; these are generated by the gear. The analysis of the signal from microphone 16 highlights that the influence of the noise produced by the turbo engines and gear is considerably reduced; instead a tonal component is identified at the frequency of 100 Hz with 2 harmonics. This tonal component can correspond to the main rotor–tail rotor interaction noise.

### 5.2. Ground Results

#### 5.2.1. Part 1. Emission–Reception

**Figure 14:**
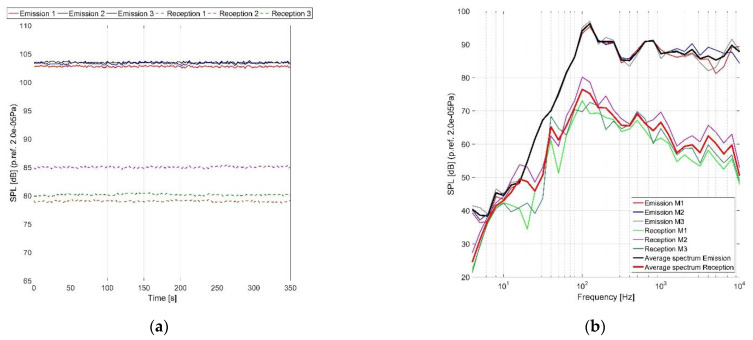
(**a**) Variation of the noise level during the measurements, (**b**) spectral analysis 1/3 octave during measurements inside and outside the helicopter.

Figure 14 shows the variation in time of the sound pressure level for each microphone during the ground tests. It can be noticed that there was no variation of the noise and the small differences of the noise between the outside microphones indicate that a diffuse acoustic field was generated on the outside helicopter structure. Inside, the differences between the acoustic pressure levels are given by the acoustic modal behavior of the cabin enclosure, where in some places low amplitudes are recorded, in others high amplitude. The differences between the overall levels of the inside microphones indicate that some doors’ weather-strips cannot assure a proper acoustic sealing so the door region is a weak point regarding the transmission of outside noise.

The 1/3 octave band spectral analysis presented above in Figure 14b shows that in the 10-3000 Hz frequency domain, the maximum difference between the acoustic spectrums is 3 dB. The difference between the maximum and the minimum of the spectral values increases after 3000 Hz to a maximum value of 5 dB, given by the acoustic directivity pattern of the loudspeakers, directivity which presents acoustic side lobes. Using the spectral analysis of the inside and outside microphones, two averaged spectra resulted which correspond to the outside (emission) and inside (reception) noise levels, Figure 14b.

**Figure 15:**
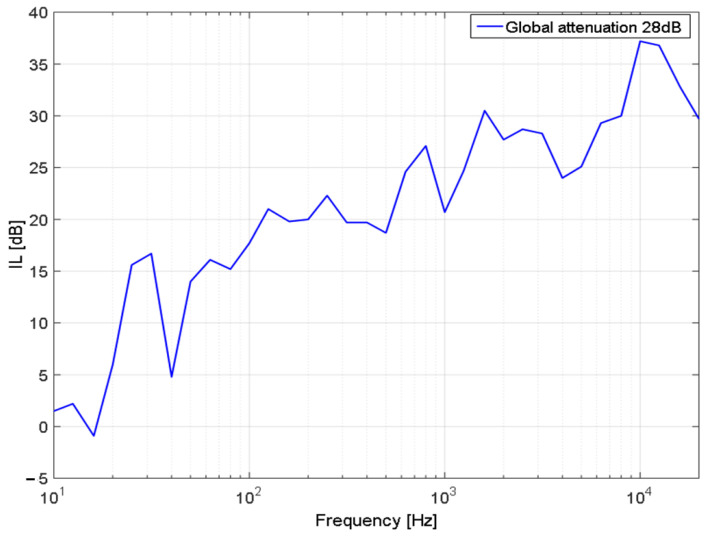
IL of helicopter structure with mounted soundproofing solution.

Based on the average pressure measured outside and inside of the helicopter, the acoustic insertion loss of the helicopter structure together with the IAR soundproofing structure (Figure 15) (IL) was computed for which an overall attenuation of 28 dB was obtained. These results can be used in a future analysis when a new soundproofing material will be implemented on the helicopter. The IL curve presents peaks and valleys that are produced by the acoustic modal response of the entire cabin; at low frequency the insulation is weak due to the thin frame of the helicopter and at high frequencies the insulation increases up to 35 dB at high frequencies.

#### 5.2.2. Part 2. Acoustic Intensity

**Figure 16:**
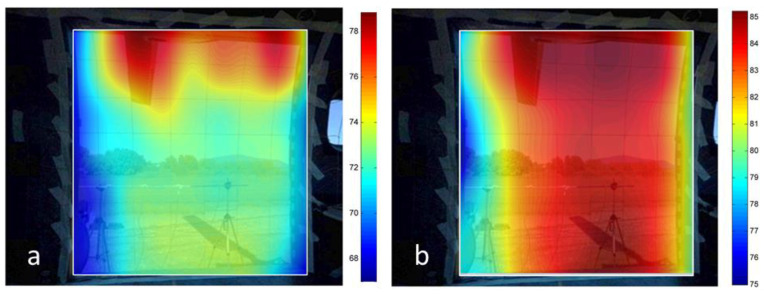
Acoustic intensity over the entire surface of the helicopter’s door: (**a**) A weighted values (dB(A)), (**b**) linear values (dB).

The acoustic intensity measurements were carried out according to SR EN ISO 9614-2:2002 [22]. As presented in Figure 6, the used measurement grid included the surface of the helicopter’s door, as well as its frame. During the measurements, a pink noise was generated, using the same configuration for the loudspeakers as in the IL determination. The results highlight the areas where acoustic leaks are located (Figure 16), which are represented with dark red. Considering the psychoacoustic factor and the A weighting, in Figure 16a one can observe that most leaks are situated in the upper part of the door, the part where the engine exhaust is situated. So, during the engines’ functioning the exhaust noise is propagating into the cabin through this region.

## 6. Conclusions

This paper presents a complete acoustic evaluation for the IAR PUMA 330 helicopters with in-flight tests and tests performed on the ground. 

One innovative aspect of this paper, compared to other similar articles, is the implementation of a procedure for evaluating the sound insulation of the helicopter structure using a set of speakers located outside the helicopter that generates a diffuse sound field. The second important aspect of this study is the identification of acoustic leaks in the area of the door sealing elements.

The acoustic measurements performed during the flight highlighted that in the area situated under the transmission gear, higher noise levels were measured. The noise levels decrease by 4.8 dB in the pilots’ cabin and in the other direction noise decreases by 9.3 dB(A). Thus, it is noted that the sound pressure levels during flight vary from 97.2 dB(A) on the helicopter tail to 106.5 dB(A) under the transmission gear. Based on all equivalent sound pressure levels the average sound level was computed, having a peak value of 106 dB at 2 kHz. The correlation between the flight data and the noise shows that the pitch is the main parameter that influences the inside noise by almost 5 dB(A). The ground test highlighted that the acoustic leaks are caused by the doors’ weather-strips and this must be changed or a new door seal system must be designed. Considering that the noise levels are different inside the helicopter, different soundproofing structures can be used to optimize the total mass of these structures. Under the transmission gear a heavier acoustic soundproofing material can be used to obtain a higher attenuation and in other areas, except the back of the helicopter where because of the existing low noise there is no need for acoustic treatment, a lighter structure can be used in comparison with the one used. 

Inside acoustic evaluation helicopter measurements are quite rare in the literature. This paper can also provide an available data set and reference for researchers in further investigations.

## Figures and Tables

**Table 1:** The min & max values during the flight.

Parameter	Min	Max
Altitude [m]	464	1031
Engine 1 speed [%]	0	92.8
Engine 2 speed [%]	0	95.8
Long true air speed [m/s]	0	237
Pitch [deg.]	−2.2	2.5

**Table 2:** The averaged overall level for each measuring zone.

Zone	1	2	3	4	5	6	7
LAeq dB(A)	101.7	104.7	106.5	105.9	103.6	99.7	97.2

## Data Availability

The datasets used and analyzed during the current study are available from the corresponding author on request.
